# 1,2,3,4,6-Pentagalloyl Glucose, a RBD-ACE2 Binding Inhibitor to Prevent SARS-CoV-2 Infection

**DOI:** 10.3389/fphar.2021.634176

**Published:** 2021-03-04

**Authors:** Rui Hong Chen, Li Jun Yang, Sami Hamdoun, Sookja Kim Chung, Christopher Wai-kei Lam, Kai Xi Zhang, Xiaoling Guo, Chenglai Xia, Betty Yuen Kwan Law, Vincent Kam Wai Wong

**Affiliations:** ^1^ Dr. Neher’s Biophysics Laboratory for Innovative Drug Discovery, State Key Laboratory of Quality Research in Chinese Medicine, Macau University of Science and Technology, Macau, China; ^2^ Department of Pharmaceutics, Faculty of Pharmacy, University of Khartoum, Khartoum, Sudan; ^3^ Faculty of Medicine, Macau University of Science and Technology, Macau, China; ^4^ Foshan Maternal and Child Health Research Institute, Foshan Women and Children’s Hospital Affiliated to Southern Medical University, Foshan, China

**Keywords:** 1, 2, 3, 4, 6-pentagalloyl glucose, RBD-ACE2 inhibitor, SARS-CoV-2, COVID-19, viral infection

## Abstract

The outbreak of SARS-CoV-2 virus caused more than 80,155,187 confirmed COVID-19 cases worldwide, which has posed a serious threat to global public health and the economy. The development of vaccines and discovery of novel drugs for COVID-19 are urgently needed. Although the FDA-approved SARS-CoV-2 vaccines has been launched in many countries recently, the strength of safety, stringent storage condition and the possibly short-term immunized efficacy remain as the major challenges in the popularity and recognition of using vaccines against SARS-CoV-2. With the spike-receptor binding domain (RBD) of SARS-CoV-2 being responsible for binding to human angiotensin-converting enzyme 2 receptor (hACE2), ACE2 is identified as the receptor for the entry and viral infection of SARS-CoV-2. In this study, molecular docking and biolayer interferometry (BLI) binding assay were adopted to determine the direct molecular interactions between natural small-molecule, 1,2,3,4,6-Pentagalloyl glucose (PGG) and the spike-RBD of the SARS-CoV-2. Our results showed that PGG preferentially binds to a pocket that contains residues Glu 340 to Lys 356 of spike-RBD with a relatively low binding energy of -8 kcal/mol. BLI assay further confirmed that PGG exhibits a relatively strong binding affinity to SARS-CoV-2-RBD protein in comparison to hACE2. In addition, both ELISA and immunocytochemistry assay proved that PGG blocks SARS-CoV-2-RBD binding to hACE2 dose dependently in cellular level. Notably, PGG was confirmed to abolish the infectious property of RBD-pseudotyped lentivirus in hACE2 overexpressing HEK293 cells, which mimicked the entry of wild type SARS-CoV-2 virus in human host cells. Finally, maximal tolerated dose (MTD) studies revealed that up to 200 mg/kg/day of PGG was confirmed orally safe in mice. Our findings suggest that PGG may be a safe and potential antiviral agent against the COVID-19 by blockade the fusion of SARS-CoV-2 spike-RBD to hACE2 receptors. Therefore, PGG may be considered as a safe and natural antiviral agent for its possible preventive application in daily anti-virus hygienic products such as a disinfectant spray or face mask.

## Introduction

The worldwide pandemic Coronavirus disease 2019 (abbreviated as COVID-19) is a viral sickness caused by the severe acute respiratory syndrome coronavirus 2 (SARS-CoV-2), which has posed a serious threat to global public health and the economy (Paules et al., 2020; Zhao et al., 2020). Over the past 20 years, two highly pathogenic human coronavirus (HCoVs) including severe acute respiratory syndrome coronavirus (SARS-CoV) and Middle East respiratory syndrome coronavirus (MERS-CoV) emerging from animal reservoirs, have led to global epidemics with high morbidity and mortality (Paules et al., 2020). While MERS-CoV does not transmit easily between humans unless they are closely contacted with each other (Zaki et al., 2012), SARS-CoV-2 is more infectious than the above two highly pathogenic coronaviruses and can be transmitted *via* either asymptomatic or presymptomatic infection. As of December 30, 2020, the coronavirus has rapidly spread to 222 countries with 80,155,187 confirmed cases and 1,771,128 deaths all over the world according to the report of World Health Organization (WHO). Typical symptoms of all these infections include fever, dyspnea, muscle ache, dry cough, sore throat and diarrhea. As the disease progressed, bilateral pneumonia, multiplemottling and ground-glass opacity in transverse chest x-ray and CT image are observed (Cui et al., 2020). Although veklury (remdesivir) is the first approved drug for COVID-19 treatment in United States (Lamb, 2020), patients with COVID-19 treated with veklury experienced severe side effects including multiple organ dysfunction syndrome, septic shock, acute kidney injury and high blood pressure. On the other hand, since the approval and the start of the vaccination program in different countries, allergic reactions and side effects including cough, loss of appetite, vomiting and diarrhea were reported with suspected correlation with the vaccine. Up to now, effective clinical treatments or prevention strategies for the highly pathogenic SARS-CoV-2 still cannot meet the demand of the huge increasing number of infected patients. Furthermore, many countries including United Kingdom, Australia and Japan have notified the mutations of coronavirus with higher contagious and pathogenic properties (Li et al., 2020), suggesting that the developed vaccine may be not effective in preventing the pandemic of COVID-19.

SARS-CoV-2 is a positive chain enveloped β-coronavirus. Similar to SARS and MERS viruses, its genome can encode non-structural proteins, structural proteins and helper proteins (Naqvi et al., 2020). Non-structural proteins include 3-chymotrypsin like protease (3CLpro), papain like protease (PLpro), helicase and RNA-dependent RNA polymerase (RdRp), whereas viral spike (S) protein is the structural protein. Among them, 4 non-structural proteins are the key enzymes for virus proliferation and replication, and S proteins are essential for virus entry through membrane receptor interaction with host cells (Shang et al., 2020). During infection, the S protein is cleaved into the N-terminal S1 subunit and C-terminal S2 subunit by host proteases such as TMPRSS2 (Simmons et al., 2013). S1 and S2 comprise the extracellular domain and a single transmembrane helix and mediate receptor binding and membrane fusion, respectively (Hoffmann et al., 2020). S1, which consists of the N-terminal domain (NTD) and the receptor binding domain (RBD), is critical in determining tissue tropism and host ranges (Wrapp et al., 2020). The RBD is responsible for binding to angiotensin-converting enzyme 2 (ACE2), previously identified as the cellular receptor for SARS-CoV (Chi et al., 2020a), while the function of NTD is not well understood. While these 5 proteins as mentioned above are considered to be important targets for discovery of anti-viral drugs, the SARS-CoV-2 Spike protein–targeting small molecules with potent neutralizing activity are a focus in the development of therapeutic interventions for COVID-19. Many studies reported the functions and structures of SARS-CoV-2–neutralizing antibodies that target the RBD and inhibit the association between the spike protein and ACE2 (Chi et al., 2020a; b;Wang et al., 2020).

1,2,3,4,6-O-Pentagalloylglucose (PGG) is a natural polyphenolic compound isolated from many traditional medicinal herbs, such as *Paeonia lactiflora* pall, *Sanguisorba officinalis* L and *Mangifera indica* (Lee et al., 2006; Liu et al., 2011; Kim et al., 2020). It has been reported to inhibit a variety of viruses although whether it can inhibit coronaviruses are not known. PGG plays a major role in inhibition the 3′-processing of HIV-1 integrase in HIV disease (Ahn et al., 2002). It can also reduce the viral HBsAg expression, a key protein in HBV DNA released for Hepatitis B virus (HBV) (Lee et al., 2006). For hepatitis C virus (HCV), PGG efficiently blocks the entry of HCV to host cells during the viral attachment (Behrendt et al., 2017). In addition, PGG shows anti-influenza-virus activity, through reducing plasma membrane accumulation of nucleoprotein at the late stage of the replication cycle and inhibit progeny virus release from the infected cells (Liu et al., 2011). Although PGG did not disrupt the integrity of the virus directly, it participates in blocking viral entry, replication and offspring release. Natural small-molecules have been playing a significant role in the prevention and treatment of emerging respiratory infectious diseases such as H1N1 influenza (Notka et al., 2004). Silico screening has identified the potential active components from Chinese herbal medicines which may inhibit 2019 novel coronavirus (Zhang et al., 2020). In the present study, molecular docking was adopted to simulate the interaction between the three-dimensional structure and interaction of the Spike-RBD and PGG. Besides, the binding of PGG to RBD using Bio-layer interferometry (BLI) and Enzyme linked Immunosorbent Assay (ELISA) were validated. Finally, by using the RBD-pseudotyped lentivirus, our results have confirmed that PGG effectively inhibited the binding and infection of virus in ACE2 overexpressing human host cells. Therefore, with the continuous evolutionary change of the virus, while the development of novel antiviral drugs and specific vaccines are still remaining the major research focus of the world, specialized personal hygiene and protective products should also be developed or modified not only for the better prevention of the disease, but more convenient and safety daily usage. For example, although most of the alcohol-based sanitizers or disinfectants available in markets can kill virus effectively, the safety risks should also be considered with its flammable nature during its usage or storage especially in the public transportations. In addition, excessive usage of bleach-based disinfectants also possess hazardous side effect to the human and the environment. Therefore, our results have discovered a non-alcoholic, safe and natural antiviral natural agent for its potential use as the active ingredient in the disinfectant spray or facial mask for neutralizing the viral spike-RBD to prevent SARS-CoV-2 infection.

## Materials and Methods

### Target and Ligand Preparation

The RBD/ACE2-B0AT1 complex of SARS-CoV-2 was downloaded from the protein data bank (ID 6M17). The target was prepared using UCSF Chimera. To isolate the receptor binding domain (RBD) amino acid chains representing the sodium-dependent neutral amino acid transporter B (0)AT1, angiotensin-converting enzyme 2 (ACE2) and the second receptor binding domain were removed. Additionally, all non-standard residues (water, N-acetyl glucosamine and zinc) were also deleted. The PDB file was then loaded and processed using Flare (Cresset, version 3.0) software. Hydrogens were added and optimal ionization states were assigned for each residue. To maximize hydrogen bond interactions and minimize steric strain, the spatial positions of polar hydrogens were optimized. The orientation of His, Asn and Gln residue side chains were optimized. Unresolved side chains were detected and reconstructed. Finally, the processed target was saved in PDB format. PGG was downloaded from Pubchem (CID 65238) in SDF format. Energy minimization and conversion to mol2 file format was performed using OpenBabel software.

### Molecular Docking

In order to predict the preferred binding pocket of corilagin on the RBD, blind docking was performed using SwissDock, a free web-based docking program. SwissDock generates all possible binding modes for the ligand under study (Grosdidier et al., 2011). The most favourable binding modes are generated and clustered at a specified pocket. All clusters were visualized with UCSF Chimera. A cluster is a predicted binding pocket on the target protein and each cluster was inspected for interacting amino acids. To determine the best pose at the predicted binding pocket, site specific docking was performed using Flare (Cresset, version 3.0) software. PGG in SDF file format was loaded and processed using default settings. The grid was selected to include the predicted binding pocket identified from the SwissDock results. The best binding pose of compound was selected for further analysis.

### Molecular Dynamics Simulation

It is essential to understand the stability of the interaction of PGG with the SARS-CoV2 RBD to further evaluate the binding affinity. The top binding pose of PGG with SARS-CoV2 RBD from the molecular docking results was subjected to MD simulation using GROMACS package version 2020.3. The topology of SARS-CoV2 RBD protein was generated using the CAHRMM36 force field. The selected binding pose of PGG was converted to Mol2 file format using Avogadro software. The topology of PGG was generated using the CGenFF server. The protein-ligand complex was generated and then solvated in a dodcahedral water box using an explicit SPC water model. The system was neutralized by adding appropriated counter ions. To minimize the energy, the system was allowed to converge at the tolerance of 1,000 kJ·mol^−1^· nm^−1^ with 500 steps of steepest descent. The system was then equilibrated in two phases. The first phase was implemented for NVT equilibration at 300 K. The second phase was performed for NPT equilibration at 1 bar pressure. The MD simulation was executed to 10 ns time. Root mean-squared deviation (RMSD) and interaction energy were stored in the trajectory for every 1 ps and were analyzed using Grace Software.

### Biolayer Interferometry

ACE2-His tag protein (Sino Biological, China) was immobilized onto the Ni-NTA probes (Fortebio, United States). SARS-CoV-2 RBD peptide (Sino Biological, China) were diluted to the different concentrations from 0.625 to 10 μg/ml. After a 60-s washing and baseline step with PBS containing 2% DMSO (Sigma, United States), respectively, biosensor tips were immersed into the wells containing SARS-CoV-2 RBD peptide with serial dilutions and allowed to associate for 300 s, followed by a dissociation step of 300 seconds. The KD value was calculated using a 1:1 binding model in Data Analysis Software 9.0 (Fortebio, United States). PGG were diluted to the different concentrations from 100 to 3.13 μM with PBS and purified SARS-CoV-2 RBD peptide (Sino Biological, China) were conjugated with biotin using EZ-Link™ Sulfo-NHS-Biotin (Genemore, China) following the manufacturer’s protocol. Then, the biotinylated SARS-CoV-2 RBD peptide were immobilized onto Super Streptavidin (SSA) biosensors (Fortebio, United States). The following experiments were as described above.

### Enzyme-Linked Immunosorbent Assay

Nickel-coated 96 well white microplates (Bioscience, United States) were coated with 1 μg/ml ACE2-His-tag protein (Bioscience, United States) following the manufacturer’s protocol. Serial dilutions of PGG were added to wells designated “Test Inhibitor” and 2% DMSO in inhibitor buffer were added to wells as “Blank” and incubated at room temperature for 1 h with slow shaking. 1 ng/uL SARS-CoV-2 RBD protein were added to wells labeled “Positive Control” and incubated at room temperature for 1 h with slow shaking. After washing with 1× Immuno Buffer 1, the plates were incubated using Blocking Buffer 2 at room temperature for 10 min. After washing with 1× Immuno Buffer 1, secondary horseradish peroxidase (HRP)-labeled antibody 1 was added at the dilution of 1:1,000 with blocking buffer 2 and incubated at room temperature for 1 h with gentle shaking. After washing, ELISA ECL substrate solution was added to the microplate. The microplate was immediately read by a luminometer (Tecan, Männedorf, Switzerland) capable of reading chemiluminescence. The data was analyzed using GraphPad Prism 7.0. Quantification bar chart represents the ELISA results from 5 independent experiments.

### Cytotoxicity Assay

Cell viability and the half maximal inhibitory concentration (IC_50_) were examined by 3-(4,5-dimethylthiazol-2-yl)-2,5-diphenyltetrazolium bromide (MTT) assay. PGG was dissolved in DMSO at a final concentration of 100 mmol/L and stored at −20°C before use. Briefly, Beas-2B, LO2 and HEK 293 cells were seeded in 96-well plates and then exposed to the tested compound at different concentrations or DMSO as a control for 72 h, respectively. Subsequently, 10-μL MTT was added to each well for 4 h followed by the addition of 100-μL solubilization buffer (10% SDS in 0.01 mol/L HCl) and overnight incubation. Absorbance at A570 nm was measured to cells viability on a plate reader (Tecan, Männedorf, Switzerland). The percentage of cell viability was calculated by the formula: Cell viability (%) = (A_treated_ –A_background_)/(A_control_ – A_background_).

### Immunocytochemistry Assay

For immunofluorescence analysis, HEK293 cells. cells were cultured in 10 cm culture dish and transfected with ACE2/EGFP construct (Vectorbuilder Inc.) for 24 h, 1×10^5^ transfected cells were seeded on the cover glasses in the 24-wells plate. The next day, PGG and SARS-CoV-2-RBD-mFc protein (Sino Biological, China) were pre-incubated for 30 min, then added into the cells and incubated for another 40 min. Cells were washed 3 times with PBST and fixed with 4% PFA for 10 min. Subsequently, cells were washed for 3 times, and then blocked in PBS containing 3% BSA for 30 min. After that, the fixed cells were incubated with goat anti-mouse IgG Fc TRITC antibody (Invitrogen Inc.) for 2 h. Cells were washed for 5 min with PBST for 3 times. The cover glasses were mounted with FluorSave reagent (Calbiochem, United States). The cells were imaged by Leica SP8 confocal microscope. For semi-quantitative determination, fluorescence images were analyzed by the Image J, and the numbers of TRIC-positive cells for each well were counted to represent infection performance. The reduction (%) in the number of TRIC-positive cells in RBD-treated wells compared with that in un-treated control wells were calculated to show the neutralizing potency.

### Pseudovirus-Based Viral Infection Assay

HEK 293 cells were seeded in 100 mm culture dish and cultured overnight. The cells were transfected with human ACE2/mCherry construct (Vectorbuilder Inc.) for 24 h. After that, 1×10^5^ transfected cells were seeded on the cover glasses of 24-well plate. The next day, PGG, RBD-pseudotyped lentivirus (Vectorbuilder Inc.) and polybrene (Vectorbuilder Inc.) were premixed in blank medium, and then added into the cells. After 12h, the medium was replaced with fresh FBS-medium and continually incubated for 48 h. The cells were then washed 3 times with PBST and then fixed with 4% PFA. The cover glasses were mounted with FluorSave reagent (Calbiochem, United States). The cells were imaged by Leica SP8 confocal microscope. For semi-quantitative determination, cells were cultured as described before. Briefly, a standard calibration curve of viral infection using 46.6, 37.3, 23.3, 4.66, 0 ×10^6^ TU of RBD-pseudotyped lentivirus were established. 3 points of cells fluorescence intensity for every titer of viral infection were calculated by Image J. Calibration curve was established for the evaluation of virus inhibition. Quantification bar chart represents the data from 5 independent experiments.

Viral infection formula: y = 286200x - 98,383, R^2^ = 0.9729.

### Maximum-Tolerate Dosage Study in C57BL/6 Mice

Male C57BL/6 mice at the age of 6–8 weeks were obtained from SPF (Beijing) Biotechnology Co., Ltd. All experiments were carried out in accordance with the “Institutional Animal Care and User Committee Guidelines” of the Macau University of Science and Technology. To determine the maximum-tolerate dosage of PGG, mice were randomly divided into 3 groups, which are control group (n = 2), low dose and high dose treatment groups (n = 4). PGG was dissolved in sterilized water contained 1% DMSO. Mice from the treated group were orally administered with 100 mg/kg or 200 mg/kg PGG, whereas the control group mice were received same volume of sterilized water contained 1% DMSO for 7 consecutive days. The health condition of mice was monitored based on their body weight and vital organs weight changes. Vital organs including liver, spleen and kidney were collected after scarification.

### Data Statistical Analysis

All experiments were repeated for at least 3 times. Results are presented as mean ± S.D. All statistical analyses were performed using Prism 6 software (GraphPad Software Inc., San Diego, CA, United States). Statistical significance among multiple group comparisons were determined by one-way analysis of variance (ANOVA). *P* value < 0.05 was considered statistically significant.

## Results

### PGG Binds and Interacts With Spike-RBD Domain of SARS-CoV-2 and ACE2 Receptor

**FIGURE 1:**
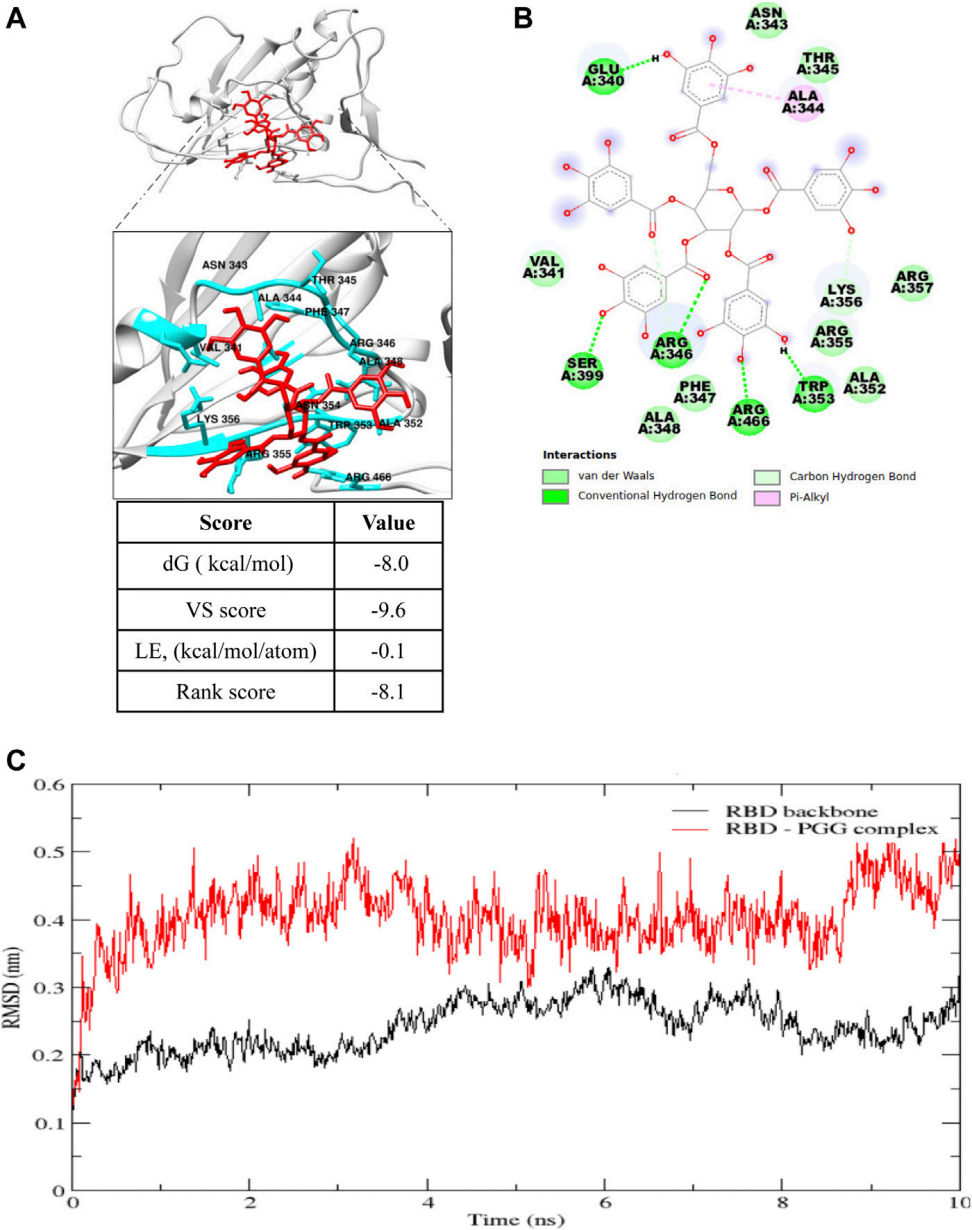
Computational docking prediction of 1,2,3,4,6-Pentagalloyl glucose (PGG). **(A)** Molecular docking result showing the best binding pose of interaction, and **(B)** the residues involved and types of interaction of PGG with the receptor binding domain of the SARS-CoV-2 spike protein. **(C)** Root mean square deviation (RMSD) plot of the SARS-CoV-2 spike protein receptor binding domain (RBD) backbone alone and in complex with PGG during the 10 ns molecular dynamics simulation.

PGG has been reported to show an anti-viral effect toward HIV, HBV, HCV and influenza-virus by blocking the viral entry and its replication (Lee et al., 2006), however, its effect toward SARS-CoV-2 remains unidentified. Therefore, the effect of PGG in interacting the viral infection of SARS-CoV-2 and thereby reducing the risk of COVID-19 was studied. To begin, the free web-based docking software SwissDock was used to predict the preferential binding site of PGG. The binding pocket with the highest number of clusters was specified as the preferential binding site (Figure 1A). As shown in Figure 1B, the preferential binding pocket for PGG involved residues Glu 340 to Lys 356. In order to specify the best binding pose more accurately, site specific docking was performed using Flare (Cresset) on slow but accurate mode. The grid was adjusted to include the predicted binding pocket. The dG, VS and LG scores were recorded for the best binding pose as shown in Figure 1A. The best binding pose was considered for analysis by molecular dynamics simulation.

In molecular dynamics simulations, the system reaches an energy minimum when a plateau is reached in the RMSD of atomic distances as a function of time. The RMSD fluctuation along with the MD simulation time, and the considerable structural change was noticed within 2 ns time as shown in Figure 1C. The average interaction energies were calculated throughout the simulation from the elelctrostatic and Lennard-Jones interaction energies. Electrostatic interaction energy was found to be -214.6 ± 19 kJ/mol and the Lennard-Jones interaction energy was found to be -154.1 ± 7.6 kJ/mol. Collectively, computational docking and molecular dynamics simulations suggest that PGG may bind and interact appropriately with Spike-RBD of SARS-CoV-2.

**FIGURE 2:**
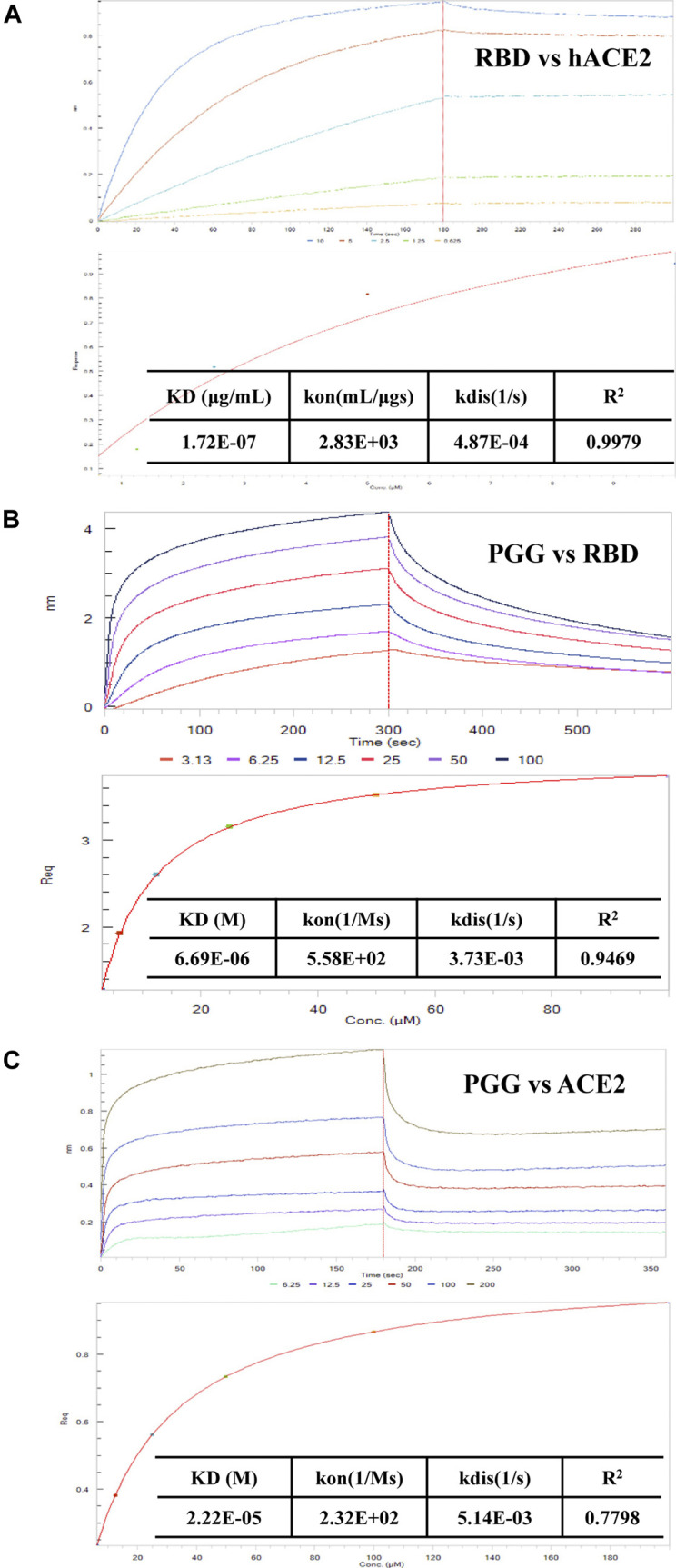
Effect of PGG on the interaction of Spike-RBD peptide and hACE2 receptor. **(A)** BLI was used to monitor the binding association of SARS-CoV-2 RBD and hACE2. **(B)** The binding kinetics and steady-state analysis of the interaction between immobilized RBD and PGG at indicated concentrations. **(C)** The binding kinetics and steady-state analysis of the interaction between immobilized ACE2 and PGG at indicated concentrations. Representative results were shown from 3 independent experiments.

To further validate the binding affinity of PGG on Spike-RBD protein, we tested the association of SARS-CoV-2 RBD and hACE2 accordingly. As shown in Figure 2A, SARS-CoV-2 RBD was dose-dependently associated with hACE2. The equilibrium dissociation constants (KD) of the interaction between SARS-CoV-2 RBD and hACE2 was 0.172 μg/ml, R2 = 0.9979 (Steady state analysis, lower panel), which confirmed that SARS-CoV-2 RBD targets and tightly binds to hACE2. After that, the Spike-RBD peptide was immobilized onto the biosensor coated with super streptavidin (SSA) *via* biotinylation and measured the binding affinity of PGG with the labeled probe using bio-layer interferometry (BLI) machine. The BLI analysis relied on the immobilization of the biotinylated Spike-RBD to the biosensor surface with subsequent exposure to various concentrations of PGG, whereby the association and dissociation phases were real-time recorded accordingly. As shown in Figure 2B, PGG was found to dose-dependently bind to Spike-RBD, whereas the binding curves of RBD suggested that a simple 1:1 binding mode occurred with R^2^ = 0.9469. Consistent with the computational docking data, the equilibrium dissociation constants (KD) of the interaction between RBD and PGG was 6.69 μM (Steady state analysis, lower panel). On the other hand, ACE2 has been shown to be a functional receptor for SARS-CoV-2 to enter host target cells (Bourgonje et al., 2020). Small-molecules adhesion on ACE2 may also affect the attachment and viral infection of SARS-CoV-2. Therefore, His-tagged ACE2 immobilized onto the biosensor coated with nickel-nitrilotriacetic acid (Ni-NTA) was used to measure the binding affinity with PGG. Concomitantly, PGG was also found to dose-dependently bind to ACE2, whereas the binding curves of ACE2 suggested that a simple 1:1 binding mode occurred with R2 = 0.7798 (Figure 2C). The equilibrium dissociation constants (KD) of the interaction between ACE2 and PGG was 22.2 μM (Steady state analysis, lower panel), suggesting that PGG may bind and interact with both viral Spike-RBD and host cells ACE2 receptor for anti-viral infection.

### PGG Directly Inhibits the Interaction of Spike-RBD Peptide and ACE2 Receptor

**FIGURE 3:**
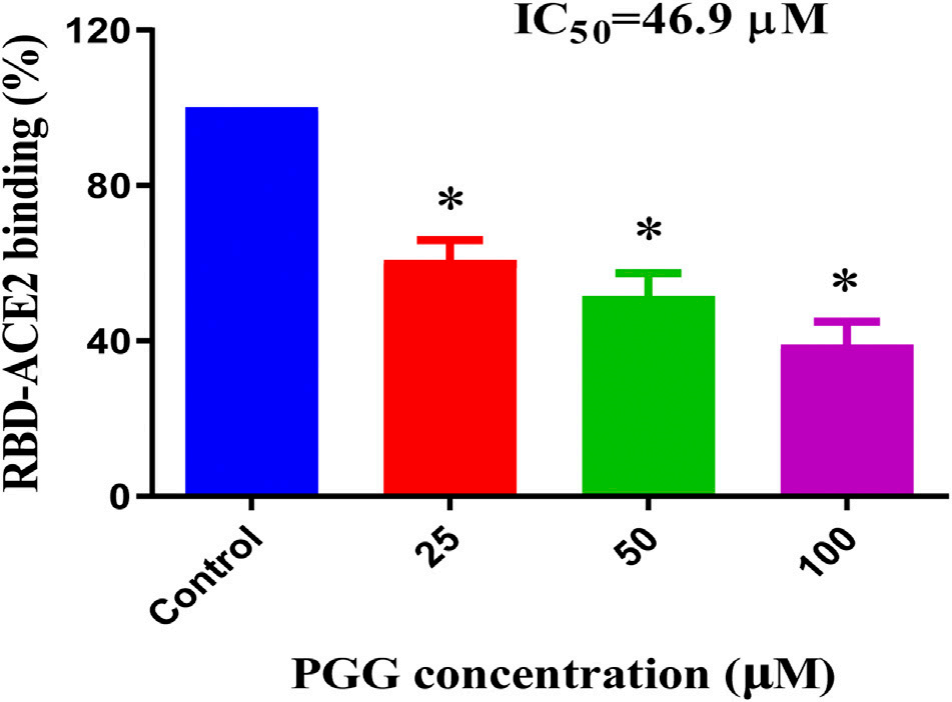
PGG reduces the Spike-RBD and ACE2 interaction. ELISA assay was adopted to determine the binding inhibitory effect of PGG to the interaction of Spike-RBD protein and ACE2 receptor. Data were expressed as mean ± S.D., n = 5; *p < 0.05, one-way ANOVA analysis.

To examine whether the binding of PGG to Spike-RBD and ACE2 can affect the interaction of Spike-RBD-ACE2, the ELISA assay with immobilization of ACE2-His-tag protein on the nickel-coated 96 well white microplates was adopted. The inhibition (%) in the optical density of PGG-treated wells compared with that in un-treated control wells were calculated to obtain the half maximal inhibitory concentration (IC_50_). As shown in Figure 3, PGG dose-dependently blocked the binding of Spike-RBD peptide to ACE2 at an IC_50_ of 46.9 μM, suggesting the possible inhibitory effect of PGG on the fusion between the viral Spike-RBD and host cells ACE2 receptor.

### PGG Suppresses the Binding and Infection of Recombinant RBD Pseudovirus in Human ACE2 Overexpressing Cells

**FIGURE 4:**
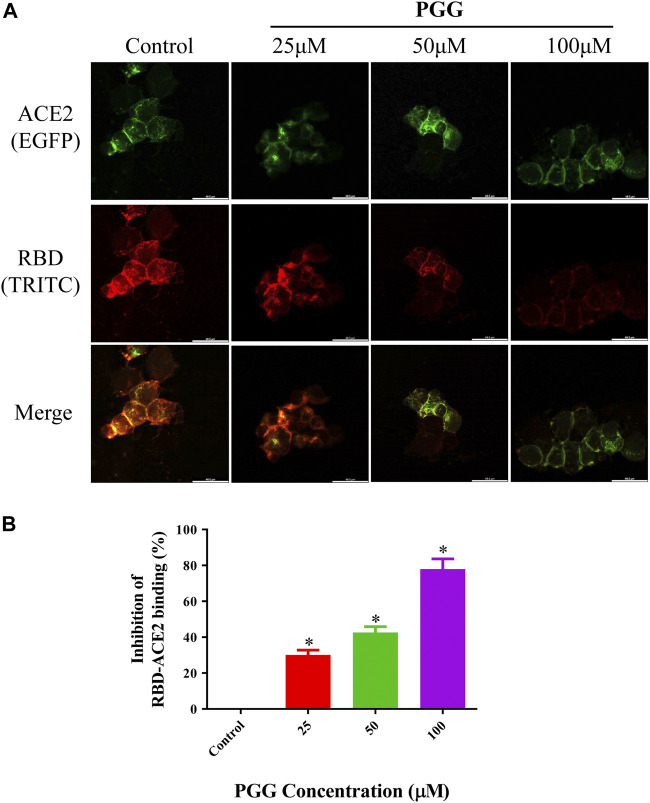
PGG suppresses the binding of Spike-RBD on ACE2 receptor in HEK293 cells. **(A)** HEK293 cells were transiently transfected with hACE2-EGFP (green). After 24 h, the cells were incubated with supernatant containing mFc-tagged SARS-CoV-2-RBD with or without PGG (25–100 μM) for 40 min. The cells were subsequently fixed and detected with mouse IgG Fc TRITC antibody (red). All images were captured by confocal microscopy using a Leica SP8 (×40 oil immersion objective lens). **(B)** Images of Spike-RBD-ACE2 binding intensity were quantified by ImageJ. Data were expressed as mean ± S.D., n = 3; *p < 0.05, one-way ANOVA analysis.

**FIGURE 5:**
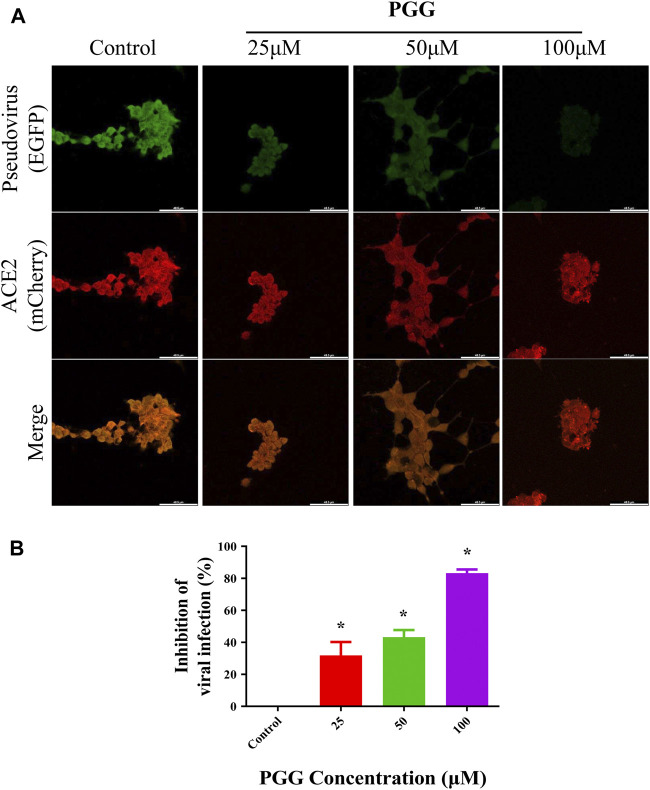
PGG inhibits the infection of S-pseudotyped lentivirus in human ACE2 overexpressing cells. **(A)** HEK293 cells were transiently transfected with hACE2-mCherry (red). After 24 h, the hACE2 overexpressing cells were infected by RBD S-pseudotyped lentivirus (green) for 12 h. The infected cells were then replaced with fresh medium and continually incubated for 48 h. All images were captured by confocal microscopy using a Leica SP8 (×40 oil immersion objective lens). **(B)** Images of RBD S-pseudotyped lentivirus infection intensity were quantified by ImageJ. Data were expressed as mean ± S.D., n = 5; *p < 0.05, one-way ANOVA analysis.

To visualize the Spike-RBD binding inhibitory effect and antiviral infectious potency of PGG *in vitro*, immunocytochemistry on RBD binding and RBD-pseudotyped lentivirus infection assay were conducted. As shown in Figure 4, mouse Fc (mFc)-fused SARS-CoV-2 Spike-RBD protein and hACE2-EGFP transfected cells were co-localized in untreated control, indicating the perfect binding of Spike-RBD on cell surface ACE2 receptor. Addition of PGG dose-dependently suppressed the binding and co-localization of Spike RBD on hACE2 (Figure 4A), indicating that the binding of PGG onto Spike-RBD protein prevented the fusion of Spike-RBD-hACE2 (Figure 4B). The inhibitory potency of PGG on viral infection was assessed by using the SARS-CoV-2 S-pseudotyped lentivirus. Consistent with the RBD binding ICC assay, HEK cells with hACE2 (mCherry) overexpression were completely infected by the S-pseudotyped lentivirus as indicated by co-localization of mCherry and EGFP fluorescence signal (Figure 5A). Moreover, viral infection assay further revealed that PGG dose-dependently inhibited SARS-CoV-2 RBD S-pseudotyped lentivirus infection (Figure 5B) as demonstrated by weak EGFP fluorescence signal. Taken together, PGG may be considered as a relatively safe SARS-CoV-2 entry inhibitor candidate to protect human cells from viral infection.

### PGG Shows Relative Low Cytotoxic Effect in Human Normal Cells and Exhibits Non-Observable Toxic Effect in C57BL/6 Mice

**FIGURE 6:**
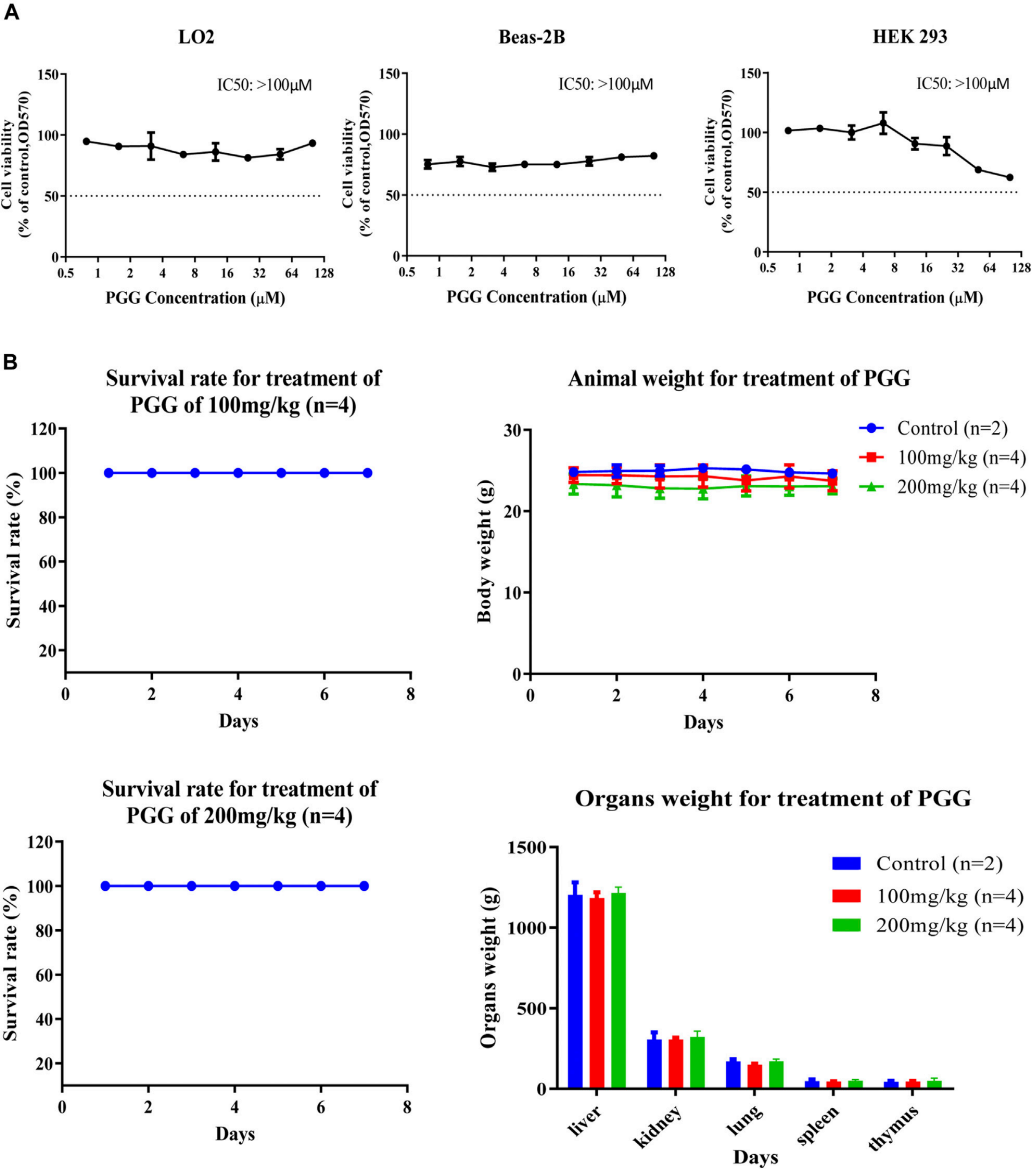
PGG exhibits no cytotoxicity and toxicity on normal cell lines and C57BL/6 mice. **(A)** PGG exhibited relative low cell cytotoxicity towards three human normal cells (LO2, BEAS-2B and HEK293). Cell cytotoxicity was measured by MTT assay. Cells were seeded and treated with PGG from 0 to 100 μM for 72 h incubation. Then, 10 μL of MTT was added to each well. After 4 h incubation, 10% SDS in 0.01 mol/L HCl was added. After 24 h later, optical density was measured by plate reader at 570 nm. **(B)** Study on the sub-chronic lethal dose of PGG. C57BL/6 mice were orally administrated with 100 or 200 mg/kg/day of PGG for consecutive 7 days, the survival, body weight and organs weight of mice were monitored and recorded. Representative results were shown as mean ± S.D. from 3 independent experiments. IC_50_ values were calculated.

To evulate the cytotoxicity of PGG, 3 human normal cell lines including lung epithelial cells (Beas-2B), normal liver hepatocytes (LO2) and human embryonic kidney 293 cells (HEK 293) were treated with PGG from 0 to 100 μM by MTT assay. Of note, PGG showed no significant cytotoxic effect on these human normal cell lines for a concentration of up to 100 μM (Figure 6A). To further investigate the *in vivo* toxic effect of PGG, maximum-tolerate dosage (MTD) of PGG was evaluated in C57BL/6 mice. As shown in Figure 6B, mice orally administrated with 100 mg/kg/day or 200 mg/kg/day of PGG indicated no toxic or harmful effect to animals as revealed by a survival rate of 100 %, no decline in body weight and organs weight after a 7-day treatment course.

## Discussion

Due the outbreak of COVID-19 at December 2019, well accepted preventive vaccine or antiviral therapeutic strategy are urgently needed to combat this deadly virus. With the long history of Chinese medicine in treating various infectious diseases, many herbal formulations have been shown to process protective effect in the intervention of COVID-19, and has provided a novel insight and the source of new drug discovery for the prevention and treatment of COVID-19 and its complications. For instance, Jinhua Qinggan granules was recommended for the treatment of COVID-19 patients in the medical observation period according to the “Diagnosis and Treatment Scheme for New Coronavirus Infected Pneumonia” (Cao et al., 2020). Lian Hua Qing Wen Capsule, Shuang Huang Lian Oral Liquid, and Qingfei Paidu Decoction were reported to show beneficial effects on COVID-19 (Yang et al., 2020b). In the current study, the active compound 1,2,3,4,6-penta-O-galloyl-b-D-glucose (PGG) has been found in Chinese medicinal herb like geranium (Piao et al., 2008). PGG has aroused special scientific interest due to its therapeutic potential in anti-tumor, antiviral, antimicrobial, anti-inflammatory, antidiabetic and anti-oxidant effects (Torres-León, 2017). Notably, PGG has been reported to delay the nuclear transport process of Herpes simplex virus type 1 (HSV-1) and suppressed its nucleocapsid egress by inhibiting the expression and cellular localization of pEGFP-UL31 and pEGFP-UL34 (Jin et al., 2016). Beside, PGG can also inhibit Influenza A virus (IAV) infection by interacting with the viral hemagglutinin (Liu et al., 2011). Furthermore, PGG was shown to effectively inhibit the cell entry of human respiratory syncytial virus (hRSV), which viral particles carrying F proteins are resistant to BMS-433771 or palivizumab (Haid et al., 2015). However, whether PGG exhibits inhibitory effect on coronaviruses has not been illustrated yet.

Structure based drug design has become a valuable and indispensable tool in drug discovery (Anderson, 2003). It makes use of three-dimensional structural information gathered from biological targets for studying the interaction with small-molecules. For instance, computational docking and molecular dynamics are the most frequently used methods for new drug prediction and discovery. These methods help in understanding the principles by which small-molecules recognize and interact with target macromolecules. In the present study, we adopted these 2 methods to predict the molecular interactions between PGG and the RBD of the SARS-CoV-2 protein. (Ferreira et al., 2015). We found that PGG preferentially bound to a pocket that involved residues Glu 340 to Lys 356. The results of the site-specific docking showed that the best binding pose of PGG interacts with a relatively low binding energy of -8 kcal/mol. It was found from the 10 ns molecular dynamics simulation that the selected binding pose was stable for the last 8 ns The binding interaction depended mainly on electrostatic interaction with minimal contribution of van der Waal’s interactions. This could be explained by the 7 hydrogen bonds shown in Figure 1. A recent study on the cryo–electron microscopy structure of SARS-CoV-2 spike (S) glycoprotein revealed that the RBD tightly bound with linoleic acid in 3 composite binding pockets (Toelzer et al., 2020). Interestingly, our predicted binding pocket was found to be one of these binding pockets. A similar pocket is found in the previous severely pathogenic strains severe acute respiratory syndrome, namely, coronavirus (SARS-CoV) and Middle East respiratory syndrome coronavirus (MERS-CoV). Binding of linoloic acid to this pocket was shown to stabilize the S-protein in the closed conformation and inhibit its transformation into the open conformation necessary for its binding to its receptor ACE2. It is therefore expected that PGG would irreversibly lock the S-protein in the closed conformation and interfere with its interaction with the receptor. In light of the interactions between PGG with the SARS-CoV-2-RBD *via* molecular docking, we speculated that the PGG should bind to the RBD protein with strong affinity. To test this hypothesis, we performed a real-time biolayer interferometry (BLI) assay. We confirmed that PGG has a relatively strong binding affinity to SARS-CoV-2-RBD protein. Analysis of ELISA assay validated PGG could dose dependently block SARS-CoV-2-RBD binding to hACE2 receptor. By ICC immuno-visualization, PGG was validated to block the binding of SARS-CoV-2 RBD protein to hACE2 receptor in cellular level. Of note, pre-incubation of PGG with RBD-pseudotyped lentivirus also abolished the infectious property of virus in hACE2 overexpressing HEK293 cells, which mimiced the entry of wild type SARS-CoV-2 virus in human host cells.

Owing to the strong infectivity, SARS-CoV-2 cannot be studied in most research laboratories without P3 standardized facilities, which may prohibit the development of new drugs. To solve this problem, the virus simulation experiment *in vitro* has been developed. The SARS-CoV-2 Spike-RBD protein is one of the most important protein for SARS-CoV-2 to enter cells, which mediates the attachment and fusion of virus to cells. A recent study characterized the cross-neutralizing activity of SARS-CoV-2, SARS-CoV and MERS-RBD protein in hACE2/293 cells and the results showed that SARS-RBD exhibits competitive inhibition with SARS-CoV-2-RBD, but not with MERS-RBD, indicating that SARS-CoV-2 and SARS-CoV have similar infection mechanism (Tai et al., 2020). In addition, SARS-CoV-2 Spike-RBD was used to develop a vaccine which can induce protective immunity (Yang et al., 2020a). Although SARS-CoV-2 Spike-RBD protein can be used to preliminarily determine whether drugs can inhibit viral ligands attach to hACE2 receptor, there are still huge variables in the biological level. Therefore, a pseudovirus containing SARS-CoV-2 Spike protein was developed. Similar to SARS-CoV-2 Spike-RBD protein, SARS-CoV-2 Spike pseudovirus initially played an important role in the identification of SARS-CoV-2 binding sites (Ou et al., 2020). In the development of vaccines, SARS-CoV-2 Spike pseudovirus has also become an important index to evaluate the viral inhibition *in vitro* (Hu et al., 2020). In our study, we utilized SARS-CoV-2 Spike-RBD protein and SARS-CoV-2 Spike pseudovirus for viral infection test. The results demonstrated that PGG had effect of competitive inhibition on SARS-CoV-2 Spike-RBD and inhibited 80% of RBD protein binding to hACE2/HEK-293 cells at 100 μM. Further experiments showed that PGG could inhibit SARS-CoV-2-Spike pseudovirus invasion by 85% at 100 μM, which was consistent with SARS-CoV-2-RBD protein binding test. In terms of safety issue, PGG was reported to inhibit the biofilm formation of *Staphylococcus aureus* and show no toxicity to human epithelial cells and fibroblasts (Lin et al., 2011). In addition, PGG can be used as a non-cytotoxic elastin stabilizer in the treatment of abdominal aortic aneurysm model rats (Isenburg et al., 2007). On the other hand, Feldman et al. reported that infusion of 50–60 mg PGG per rat (∼200 g body weight) resulted in a precise and lethal drop in blood pressure within 30 min, whereas 30 mg per rat did not affect blood pressure or blood glucose levels (Feldman et al., 2001). Moreover, PGG is astringent which can inhibits human salivary α-amylase, potential negative effect on starch digestion and food taste (Hofmann et al., 2006). In our study, we found that PGG had no toxic effect on the 3 normal cell lines including BEAS-2B, LO2 and HEK293 cells. Meanwhile, we observed that there was no significant weight loss in animal body or their vital organs in C57BL/6 mice, suggesting that PGG could be considered safe for external use. Taken together, these results indicated that PGG might be further developed as an effective anti-viral agent for external use, e.g., anti-viral spray to fight against the COVID-19 by blockade of the binding of spike-RBD of SARS-CoV-2 to cellular ACE2 receptors.

## Data Availability

The raw data supporting the conclusions of this article will be made available by the authors, without undue reservation.
